# Relating perturbation magnitude to temporal gene expression in biological systems

**DOI:** 10.1186/1756-0500-2-43

**Published:** 2009-03-19

**Authors:** Stephen J Callister, J Jacob Parnell, Michael E Pfrender, Syed A Hashsham

**Affiliations:** 1 Department of Civil and Environmental Engineering, Michigan State University, East Lansing, MI, 48824, USA; 2 Center for Microbial Ecology, Michigan State University, East Lansing, MI, 48824, USA; 3 Biological Separations and Mass Spectrometry, Pacific Northwest National Laboratory, Richland, WA 99352, USA; 4 Department of Biology and Ecology Center, Utah State University, Logan, UT, 84322, USA; 5 Center for Integrated BioSystems, Utah State University, Logan, UT, 84322, USA

## Abstract

**Background:**

Most transcriptional activity is a result of environmental variability. This cause (environment) and effect (gene expression) relationship is essential to survival in any changing environment. The specific relationship between environmental perturbation and gene expression – and stability of the response – has yet to be measured in detail. We describe a method to quantitatively relate perturbation magnitude to response at the level of gene expression. We test our method using *Saccharomyces cerevisiae *as a model organism and osmotic stress as an environmental stress.

**Results:**

Patterns of gene expression were measured in response to increasing sodium chloride concentrations (0, 0.5, 0.7, 1.0, and 1.2 M) for sixty genes impacted by osmotic shock. Expression of these genes was quantified over five time points using reverse transcriptase real-time polymerase chain reaction. Magnitudes of cumulative response for specific pathways, and the set of all genes, were obtained by combining the temporal response envelopes for genes exhibiting significant changes in expression with time. A linear relationship between perturbation magnitude and response was observed for the range of concentrations studied.

**Conclusion:**

This study develops a quantitative approach to describe the stability of gene response and pathways to environmental perturbation and illustrates the utility of this approach. The approach should be applicable to quantitatively evaluate the response of organisms via the magnitude of response and stability of the transcriptome to environmental change.

## Background

The cause and effect relationship between perturbation and response is routinely used to study and characterize ecosystems in terms of stability. To extend this concept, we investigated the relationship between the affect of different perturbation magnitudes on the dynamic level of transcriptional response. The establishment of a quantitative relationship between stress and response has implications for predictive capabilities related to the behavior of organisms in natural and engineered systems and can be established on many levels including community, population, proteome, and transcriptome.

Transcription is often compared by measuring the fold-change in relative expression [1,2]; however, a simple fold-change approach only accounts for one aspect of the response. Adjusting to perturbation spans a continuum of short-term stress responses, long-term acclimation, and genetic adaptation. Improved analytical tools for understanding the transcriptional mechanisms underlying variation in environmental tolerance will help address key issues such as what distinguishes generalists from specialists, and how do organisms increase tolerance [3,4].

We evaluate a standard area of displacement to determine the quantitative relationship between perturbation and transcriptional response. We selected *Saccharomyces cerevisiae *as a model organism because of its existing knowledgebase related to gene expression, particularly in high salt concentration (model stress). Our method demonstrates a linear relationship resulting in a proportional response at very high perturbation magnitudes.

## Methods

### Cell culture

*Saccharomyces cerevisiae *W303 (ATCC 200060) was grown in batch culture at 30°C in YDP medium containing 2% bacto-peptone, 1% yeast extract, and 2% glucose. Cells were harvested by centrifugation, washed, and suspended in YDP to a final OD_600 _of approximately 0.16. 5 M NaCl was added in triplicate to early log phase (OD_600 _of ~1.0) cells resulting in a final NaCl concentration of 0.5 M, 0.7 M, 1.0 M, or 1.2 M. Prior to salt addition, a 15 ml sample was collected to characterize the base-line gene expression level. During perturbation, cells were harvested at regular intervals [5] until growth resumed as indicated by three consecutive increases in OD_600_.

### RNA extraction and purification

Total RNA was extracted using the modified hot acid phenol extraction method [6]. A 5 *μ*l sub-sample was removed and reserved for RNA quantification and to check for genomic DNA contamination. All RNA extractions were performed in a dedicated hood, cleaned to reduce or destroy residual RNase activity using either a 10% bleach solution or RNase wipes (Ambion, Austin TX).

Extracted RNA was evaluated for DNA contamination using a no-RT control as described previously [7] with the following PCR conditions as follows: 94°C 2 min, 40 cycles of 94°C 0.5 min, 55°C 0.5 min, 72°C 1 min followed by a final elongation step 72°C 1 min. Amplified PCR products were detected on a 3% agarose gel. Samples containing DNA contamination digested with Turbo™ DNase (Ambion, Austin, TX) at 37°C for 30 minutes and reevaluated for DNA contamination.

### Gene selection and primer design

Sixty genes were selected for observation during osmotic shock based on whether: a) the genes exhibit significant expression to osmotic shock [5,8,9], b) the genes show connection to osmotic shock, although insignificant expression in microarray results [5], and c) the genes are connected with one of the three biochemical pathways associated with salt stress, namely gycolysis, glycerolipid, and the high-osmotic glycerol (HOG) pathway, based on the Kyoto Encyclopedia of Genes and Genomes (KEGG) database [10] (Table 1).

**Table 1:** Genes used in this study belonging to one of three osmotolerance biochemical pathways.

Gene	Description	Pathway
YAL038W	Pyruvate kinase	Glycolysis
YBR019C	UDP-glucose-4-epimerase	Glycolysis
YBR029C	Phosphatidate cytidylyltransferase (CDP-diglyceride synthetase)	Glycerolipid
YBR196C	Glycolytic enzyme phosphoglucose isomerase	Glycolysis
YCL004W	Phosphatidylglycerolphosphate synthase	Glycerolipid
YCL040W	Glucokinase	Glycolysis
YCR012W	3-phosphoglycerate kinase	Glycolysis
YDL021W	Homolog of Gpm1p phosphoglycerate mutase	Glycolysis
YDL022W	NAD-dependent glycerol-3-phosphate dehydrogenase	Glycerolipid
YDL052C	1-acyl-sn-gylcerol-3-phosphate acyltransferase	Glycerolipid
YDL142C	Cardiolipin synthase	Glycerolipid
YDL168W	Bifunctional enzyme containing both alcohol dehydrogenase and glutathione-dependent formaldehyde dehydrogenase activities	Glycerolipid
YDL235C	Phosphorelay intermediate protein	HOG
YDL243C	Putative aryl-alcohol dehydrogenase	Glycerolipid
YDR050C	Induced under stress conditions; triosephosphate isomerase	Gycolysis
YDR147W	Ethanolamine kinase	Glycerolipid
YDR380W	Phenylpyruvate decarboxylase	Glycolysis
YDR481C	Repressible alkaline phosphatase	Glycerolipid
YER026C	phosphatidylserine synthase	Glycerolipid
YER062C	DL-glycerol-3-phosphatase	Glycerolipid
YER073W	Mitochondrial aldehyde dehydrogenase	Glycerolipid
YER118C	Transmembrane osmosensor	HOG
YER178W	E1 alpha subunit of the pyruvate dehydrogenase (PDH) complex	Glycolysis
YFL056C	Putative aryl-alcohol dehydrogenase	Glycerolipid
YFR053C	Hexokinase isoenzyme 1	Glycolysis
YGL257C	Mannosyltransferase	Glycolysis
YGR007W	Choline phosphate cytidylyltransferase	Glycerolipid
YGR088W	Cytosolic catalase T	HOG
YGR170W	Phosphatidylserine decarboxylase	Glycerolipid
YGR202C	Cholinephosphate cytidylyltransferase	Glycerolipid
YGR240C	Alpha subunit of heterooctameric phosphofructokinase	Glycolysis
YHL007C	Signal transducing kinase of the PAK (p21-activated kinase) family	HOG
YHL032C	Glycerol kinase	Glycerolipid
Gene	Description	Pathway
YHR123W	sn-1,2-diacylglycerol ethanolamine- and cholinephosphotranferase	Glycerolipid
YIL014W	Alpha-1,3-mannosyltransferase	Glycerolipid
YIL147C	Histidine kinase osmosensor that regulates a MAP kinase cascade	HOG
YIL155C	Mitochondrial glycerol-3-phosphate dehydrogenase	Glycerolipid
YJL128C	MAP kinase kinase	HOG
YKL060C	Fructose 1,6-bisphosphate aldolase	Glycolysis
YKR031C	Phospholipase D	Glycerolipid
YLR006C	Cytoplasmic response regulator	HOG
YLR113W	Mitogen-activated protein kinase	HOG
YLR133W	choline kinase	Glycerolipid
YLR362W	Signal transducing MEK kinase involved	HOG
YLR377C	Fructose-1,6-bisphosphatase	Glycolysis
YML004C	Monomeric glyoxalase I	HOG
YML070W	Dihydroxyacetone kinase	Glycerolipid
YMR037C	Transcriptional activator related to Msn4p	HOG
YMR043W	Transcription factor	HOG
YMR105C	Phosphoglucomutase	Glycolysis
YMR169C	Aldehyde dehydrogenase that uses NAD+ as the preferred coenzyme	Glycolysis
YMR303C	Glucose-repressible alcohol dehydrogenase II	Glycolysis
YNL130C	Cholinephosphotransferase	Glycerolipid
YNR031C	Suppressor of Sensor Kinase (SLN1)	HOG
YOL056W	Homolog of Gpm1p phosphoglycerate mutase	Glycolysis
YOL059W	NAD-dependent glycerol 3-phosphate dehydrogenase	Glycerolipid
YOR120W	Putative NADP(+) coupled glycerol dehydrogenase	Glycerolipid
YOR374W	Mitochondrial aldehyde dehydrogenase	Glycolysis
YPL017C	Putative S-adenosylmethionine-dependent methyltransferase	Glycolysis
YPL206C	Endoplasmic reticulum protein of unknown function	Gycerolipid
YPR113W	Phosphatidylinositol synthase	Glycerolipid

Primers for each gene were designed using Primer Express™ 2.0 (Perkin Elmer Applied Biosystems, Foster City, CA). Design parameters included a T_m _from 58°C to 62°C, GC content from 45% to 55%, length from 20 bp to 22 bp, and amplicon length from 100 bp to 110 bp. Additional design parameters were set according to the manufacturer's suggestions. Specificity of primers was evaluated by gel electrophoresis of the amplified products, and analyzing dissociation curves for amplicons following quantitative real-time PCR.

### Quantitative real-time PCR

RNA samples were reverse transcribed using a High Capacity cDNA Conversion Kit™ (Applied Biosystems, Foster City, CA), purified using Qiagen PCR purification columns (Qiagen, Valencia, CA) and quantified at 260 nm. 10–20 ng of purified cDNA was added to a reaction cocktail containing the PCR buffer described earlier with 2.5% (vol/vol) SYBR™ Green I (Molecular Probes, Eugene, OR; 1000×), and 2.3 *μ*M 6-ROX (6-carboxy-x-rhodamine, Molecular Probes, Eugene, OR). PCR was performed on an ABI 7900 HT (Applied Biosystems, Foster City, CA) using the reaction conditions, minus the final elongation step, described earlier.

Calibration curves of template dilution series of a 101 bp fragment of inorganic phosphatase and a 106 bp fragment of glycerol-3-phosphate were run in triplicate for each PCR run to normalize differences in amplicon sizes. The 101 bp and 106 bp fragments corresponded to the range of amplicons bounded by the primers designed for selected genes. PCR products were purified using 10,000 MWCO spin columns (Millipore, Bedford, MA), and quantified by absorbance at 260 nm. Purified products were adjusted to the same absorbance and a 1:10 dilution series was prepared as the starting template for the standard curves. Four dilutions from each series (10^0^, 10^-2^, 10^-4^, and 10^-6^) were evaluated for linearity and agreement in cycle threshold (Ct).

### Algorithm

To evaluate gene expression patterns over time to the applied perturbations, the relative response of an individual gene was calculated as:

(1) *r*(*t*)_*i *_= (*x*_*i *_(*t*) - *x*_*i *_(0))/*x*_*i *_(0)

where, *x*_*i*_(*t*) represents the arbitrary expression for gene *i *at time *t*. Equation 1 represents the deviation of gene expression from the onset of the perturbation, *x*_*i*_(0). The aggregate relative response of all genes or pathways at a given time was calculated as the difference in the cumulative responses of genes making up the set or pathway:

(2) $$c\left(t\right)=\left(\sum_{1}^{i}x_{i}\left(t\right)-\sum_{1}^{i}x_{i}\left(0\right)\right)/\sum_{1}^{i}x_{i}\left(0\right).$$

This calculation for a given time period represents the relative response envelope of the aggregate. For the response of a gene to be included in the aggregate relative response, the gene must meet the following criteria: 1) a measured response in two of three replicates at each sampling event (five total), 2) the measured response was within the dynamic range of the standard curve used to determine its expression value, and 3) the fractional coefficient of variation among replicate measurements was less than 1. This latter criterion was included because the cumulative response is highly subject to uncertainty from propagation of random error. To quantify the total amount of displacement, the area inscribed by the response envelope was determined by curve fitting using a polynomial regression model:

(3) *y*(*t*) = *β*_0 _+ *β*_1 _*t*_1 _+ *β*_2 _*t*_2 _+ ... *β*_*k *_*t*_*k*_.

Where, *β*_*k *_are the coefficients describing the shape of the relative response and *y(t) *is the specific model for each relative response envelope from the onset of the perturbation (t = 0) to the last measurement.

## Results and discussion

**Figure 1:**
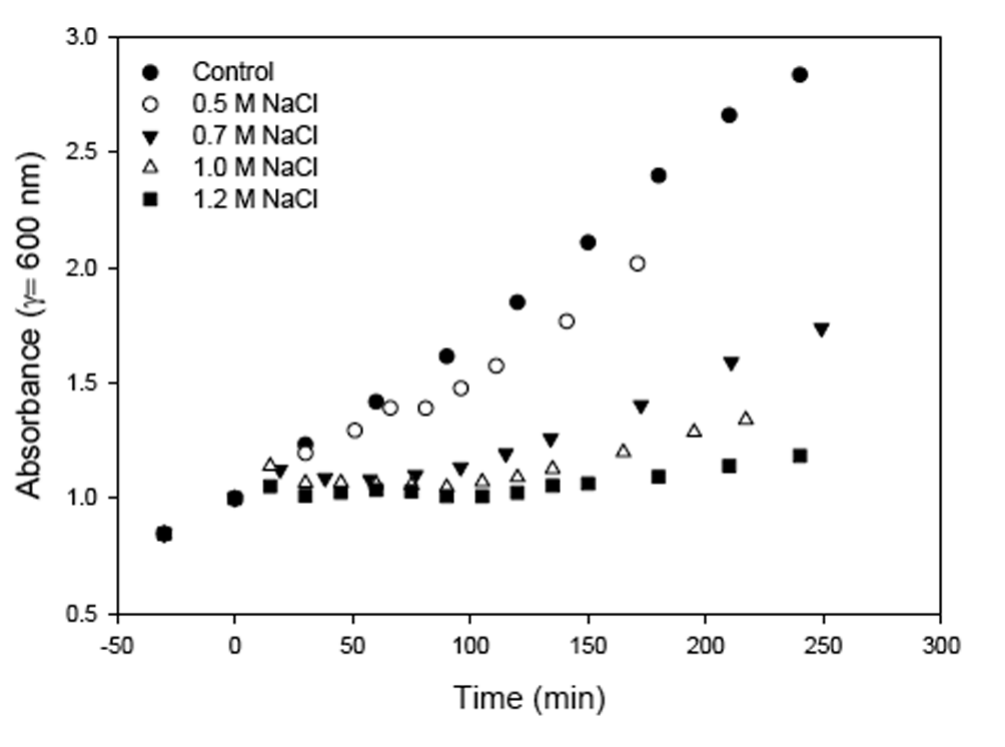
**Effect of elevated salt concentrations on the growth of *S. cerevisiae***. Growth in response to 5 press perturbations of NaCl addition to the growth medium resulting in the final NaCl concentrations indicated. *S. cerevisiae *displayed the typical behavior to NaCl addition, mainly an arrest in growth at or shortly after the onset of the perturbation followed by a period of adjustment, and then a resumption of growth at a lower rate. The lag period following addition of NaCl to a concentration of 0.5 M was barely detectable, while at the 1.2 M NaCl perturbation resumption of growth was barely noticeable, which suggested at this perturbation magnitude the cell culture had been significantly impacted by the effects of osmotic shock and NaCl toxicity.

**Figure 2:**
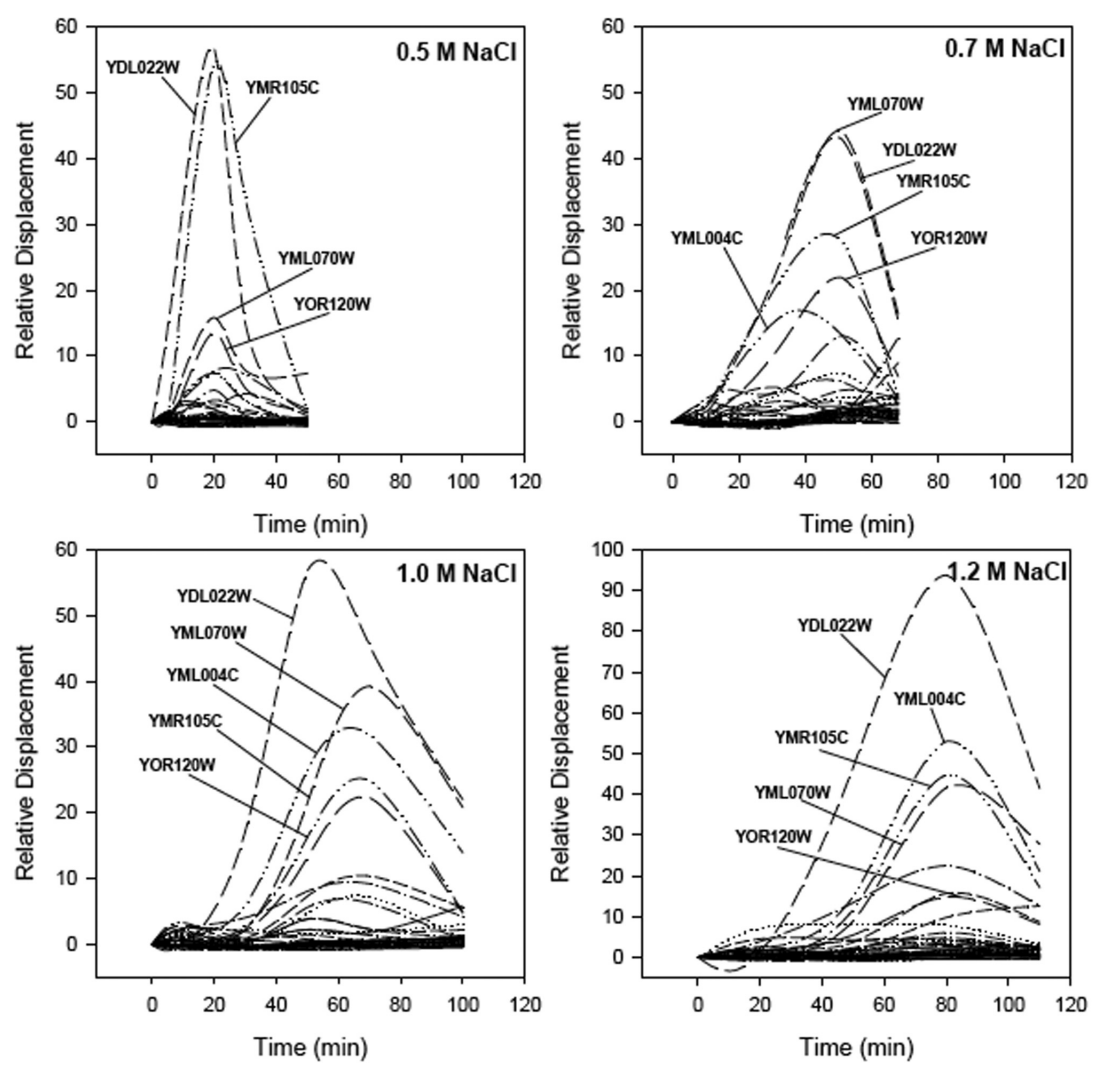
**Relative response envelopes for salt-tolerance genes in *S. cerevisiae *determined by Q-RT-PCR following perturbation by addition of: A) 0.5 M NaCl, B) 0.7 M NaCl, C) 1.0 M NaCl, and D) 1.2 M NaCl**. Three of the genes induced during salt stress (YDL022W, YML070W & YOR120W) are responsible for glycerol production and correspond to the glycerolipid pathway. Two other genes with elevated expression, YML004C and YMR105C, correspond to the high-osmotic glycerol (HOG) and glycolysis pathways, respectively.

Cell growth in response to NaCl was typified by a lag phase followed by resumption of growth at a reduced rate (Fig. 1) [11-13]. The relative displacement of the selected genes indicates increased expression followed by decreased expression for all perturbation magnitudes tested (Fig. 2) including a return of some genes to pre-perturbed levels (see Fig. 2A). While the lag phase has been hypothesized as an adjustment required for growth in new conditions [14], it remained unclear why progression through the cell cycle is arrested. By analyzing the response envelope to different salt concentrations, we found that the suspension of growth coincides with the reset to a new steady state (Figs 1 &2). The increasing lag phase and decreased growth rates at greater salt concentrations suggest that exceeding 1.2 M NaCl would result in failure to resume growth in agreement with other reported values [15-17]. Note, that while high salt concentration results in osmotic stress, high NaCl concentrations can also induce sodium toxicity; however, these results are limited to known osmotolerance mechanisms.

One of the most dominant genes in all four perturbations is *GPD1 *(glycerol-3-phosphate dehydrogenase). However, *DAK1 *(dihydroxyacetone kinase), *GLO1 *(oxidoreductase), *PGM2 *(phosphoglucomutase), and *GCY1*, (phosphatidylserine synthase) follow similar patterns. These genes, in part, produce and regulate glycerol; essential for growth under osmotic stress [18-20]. *GPD1*, *DAK1 *and *GCY1 *are thought to be co-transcribed [19]. Glycerol is synthesized from glucose, and a rate-limiting enzyme in glycerol biosynthesis is the *GPD1 *gene product, which catalyzes reduction of dihydroxyacetone phosphate to glycerol 3-phosphate [14,21,22]. Although the halometric increase of *GPD1 *and *DAK1 *has been noted [23], the increase of *GPD1 *is proportional to the perturbation up to 1.2 M NaCl, while the abundance of *DAK1 *plateaus between 0.5 and 0.7 M NaCl (see Figs. 2C &2D). *GLO1 *detoxifies a cytotoxic metabolite derived from dihydroxyacetone phosphate which increases during glycerol production [22]. *PGM2 *converts glucose-1 phosphate and glucose-6 phosphate [24] and is an important factor in glycolysis (eventually leading to glycerol production) as well as the production of other compatible solutes such as trehalose during salt stress.

**Figure 3:**
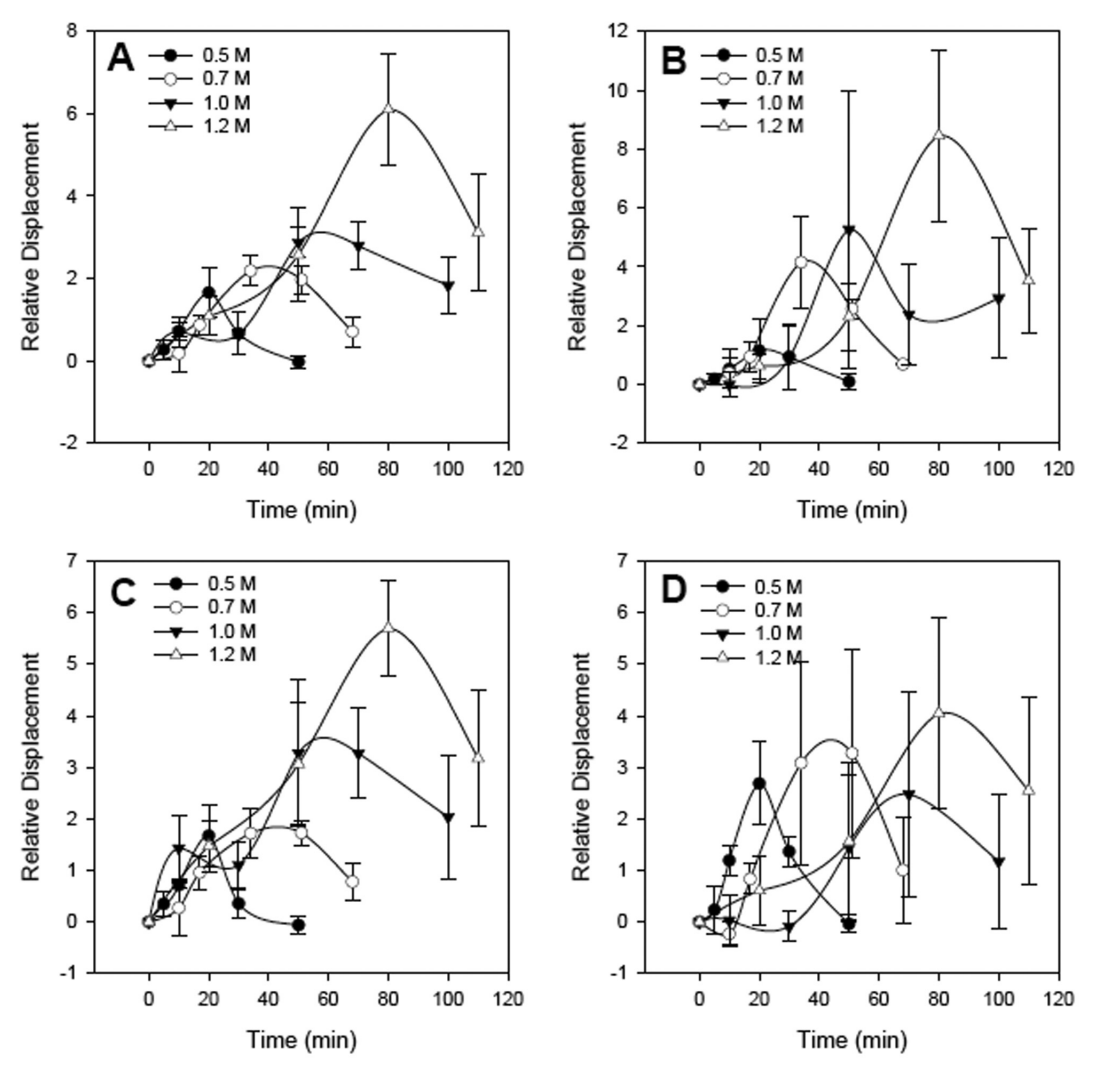
**Relative response envelopes used to calculate strain for: A) the aggregate set of all genes, B) high-osmotic glycerol (HOG), C) glycerolipid metabolism, and D) glycolysis pathways**. Relative displacement shown for the pathways was normalized by the number of genes making up each pathway. Comparison of response envelopes between pathways and the aggregate set of genes revealed different trends of behavior with increasing perturbation magnitude. These trends suggested a different contribution of each pathway to the aggregate relative response for each perturbation magnitude.

The ecological parameters, resistance and resilience were estimated from the aggregate response envelops, the cumulative displacement of all measured genes (Fig. 3A). From the response envelopes, the degree of resistance was estimated as the maximum displacement, with a larger value for this displacement indicating a lesser degree of resistance [25]. Resilience, measured as the approximate duration of the response, was ~1.4-fold less for the 0.7 M response compared to the 0.5 M response and ~3-fold less for the 1.2 M perturbation compared to the 0.5 M perturbation. Response envelopes for the individual pathways involved in osmotic stress response, the high-osmotic glycerol (HOG) (Fig. 3B) and the Glycerolipid (Fig. 3C) synthesis exhibited monotonic behavior similar to the aggregate set of genes. Contrary to the monotonic increase in response envelope associated with increased stress seen in the two pathways mentioned above, the glycolysis pathway demonstrated a greater resilience at the 0.5 M perturbation compared to the 1.2 M perturbation (Fig. 3D).

**Figure 4:**
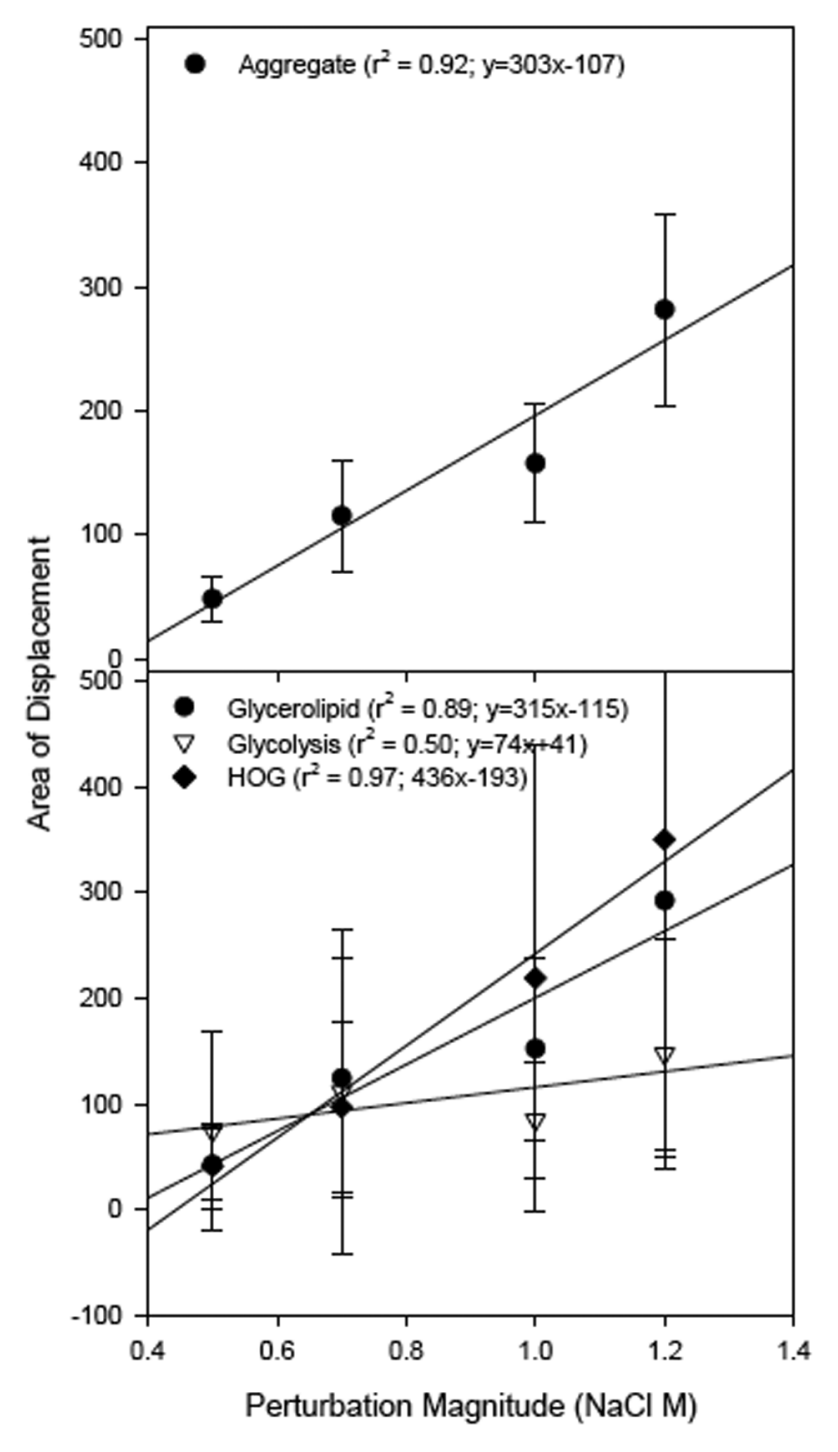
**Relative area of displacement versus perturbation magnitude for the aggregate response of all genes (top), and genes grouped by biochemical pathway (bottom)**. A linear relationship was observed between stress and response, with the highest response resulting from the 1.2 M NaCl perturbation.

The area of relative displacement increased in response to increased stress. For the aggregate of selected genes (Fig. 4A), the area of relative displacement associated with the 0.7 M stress was approximately 2.4 fold greater with respect to the 0.5 M stress. Overall, the area of displacement increased with stress and exhibited a high degree of linearity (r^2 ^= 0.92), suggesting a linear relationship between osmotic stress and transcriptional response. A linear trend in response to increased stress was also observed for the biochemical pathways (Fig. 4B) with the exception of the glycolysis pathway (r^2 ^= 0.50), where observed increase in the area of relative displacement in response to an increase in stress was not significant. The high degree of linearity for both the glycerolipid and HOG pathways is due to their direct association with osmotic stress (whereas glycolysis regulation is not solely influenced by osmotic stress). The proportional rate of increase (slope) was most dramatic for the HOG metabolism pathway and glycerolipid production. Because the area of relative displacement includes both the resistance and resilience components of stability, its larger rate of increase suggests these pathways are less stable compared to the glycolysis pathway.

The ability of a system, in this case the set of selected genes and pathways, to adjust to environmental changes has been described as resilience [26-29], and the ability to withstand the effects imposed by the changes – similar to the concept of buffering capacity – has been described as resistance [25,28]. Resilience at each perturbation magnitude (0.5, 0.7, 1.0, and 1.2 M NaCl) was determined by the time required for either the aggregate set of genes or biochemical pathway to approach pre-perturbed displacement [25] or the new steady state of *S. cerevisiae *gene expression following exposure to high salt concentrations [23]. Resistance was estimated as the maximum displacement of the response envelope [25]. In terms of the aggregate set of genes and pathways, a larger stress resulted in decreased resilience (see Figs. 2 &3) and less resistance.

## Conclusion

Although our model determines the resilience and resistance of *S. cerevisiae *genes and pathways to elevated NaCl concentrations, this approach can be applied to measure the transcriptional response (positive or negative) to a range of environmental changes. This model is designed to quantify an overall response by determining the change in expression. In the terms of strain, or transcriptome response, down-regulated and up-regulated genes can be represented as a positive response value, for instance one gene that is up-regulated and another equally down-regulated will not cancel each other out, but the response value will be amplified.

Examining the response over a prolonged exposure to stress, rather than a snapshot approach, allows the quantification of the response to increased stress. This approach allows a direct comparison of the contribution of each gene or pathway on the aggregate transcriptome. The relationship between fitness and environmental conditions is inherently a multigenic phenomenon requiring this type of an approach. Understanding the relationship between stress, response, and resulting stability at the level of the transcriptome could provide predictive capabilities for other areas of biological research, such as engineering biostimulation for de-toxification of pollutants, and as a predictive indicator for adaptation to changing environments.

## Authors' contributions

SJC and SAH designed this study, SJC collected and analyzed data, SJC, JJP and MEP interpreted data and wrote the manuscript, all authors read and approved the final draft.
